# Estrogen receptor positive breast cancers in *BRCA1 *mutation carriers: clinical risk factors and pathologic features

**DOI:** 10.1186/bcr2478

**Published:** 2010-02-11

**Authors:** Nadine Tung, Yihong Wang, Laura C Collins, Jennifer Kaplan, Hailun Li, Rebecca Gelman, Amy H Comander, Bridget Gallagher, Katharina Fetten, Karen Krag, Kathryn A Stoeckert, Robert D Legare, Dennis Sgroi, Paula D Ryan, Judy E Garber, Stuart J Schnitt

**Affiliations:** 1 Division of Hematology-Oncology, Beth Israel Deaconess Medical Center, Brookline Avenue, Boston, MA 02215, USA; 2 Harvard Medical School, Shattuck Street, Boston, MA 02115, USA; 3 Department of Pathology, Beth Israel Deaconess Medical Center, Brookline Avenue, Boston, MA 02215, USA; 4 Department of Biostatistics and Computational Biology, Dana-Farber Cancer Institute, Binney Street, Boston, MA 02115, USA; 5 Program in Oncology, North Shore Medical Center, Endicott Street, Danvers, MA 01923, USA; 6 Division of Population Sciences and Adult Oncology, Dana-Farber Cancer Institute, Binney Street, Boston, MA 02115, USA; 7 Program in Women's Oncology, Women and Infants Hospital, Dudley Street, Providence, RI 02905, USA; 8 Department of Pathology, Massachusetts General Hospital, Fruit Street, Boston, MA 02114, USA; 9 Division of Medical Oncology, Massachusetts General Hospital, Fruit Street, Boston, MA 02114, USA

## Abstract

**Introduction:**

Most breast cancers that occur in women with germline *BRCA1 *mutations are estrogen receptor-negative (ER-) and also typically lack expression of progesterone receptor (PR) and HER2 overexpression. We undertook a study to assess the clinical factors that predict for an estrogen receptor positive (ER+) breast cancer in *BRCA1 *mutation carriers and to characterize the pathologic features of these tumors.

**Methods:**

Clinical characteristics of *BRCA1 *carriers with 58 ER+ and 114 ER- first invasive breast cancers were compared. Pathologic features of *BRCA1 *ER+ cancers were compared to those of *BRCA1 *ER- cancers and to age-matched ER+ sporadic cancers.

**Results:**

*BRCA1 *carriers aged ≥ 50 at diagnosis of first invasive breast cancer were more likely to have an ER+ cancer compared to those aged < 50 (57% vs 29%, *P* = 0.005). ER+ *BRCA1 *cancers were less likely than ER- *BRCA1 *cancers to have "*BRCA*-associated" features such as high mitotic activity, geographic necrosis/fibrotic focus, and pushing margins (RR 0.06, 0.22, 0.24; *P* < 0.001, 0.02, 0.03 respectively). When compared to sporadic ER+ cancers, ER+ *BRCA1 *cancers were more often of invasive ductal type (RR 2.4, *P* = 0.03), with a high mitotic rate (RR 5.0, *P* = 0.006) and absent or mild lymphocytic infiltrate (RR 10.2, *P* = 0.04).

**Conclusions:**

*BRCA1 *carriers who are older at first breast cancer diagnosis are more likely to have ER+ tumors than younger *BRCA1 *carriers. These ER+ cancers appear pathologically "intermediate" between ER- *BRCA1 *cancers and ER+ sporadic breast cancers raising the possibility that either some ER+ *BRCA1 *cancers are incidental or that there is a unique mechanism by which these cancers develop.

## Introduction

Most breast cancers that occur in women with germline *BRCA1 *mutations are estrogen receptor-negative (ER-) and typically lack expression of progesterone receptor (PR) and human epidermal growth factor receptor (HER) 2 overexpression (so-called 'triple-negative' breast cancers) [1-8]. These *BRCA1*-associated ER-tumors are most often high-grade invasive ductal carcinomas with a high mitotic rate that frequently exhibit other characteristic pathologic features including a prominent lymphocytic infiltrate, pushing or circumscribed margins, and geographic areas of necrosis or a central fibrotic focus [3,9,10]. In addition, these tumors often express 'basal' biomarkers and most cluster within the 'basal-like' group in gene expression profiling studies [7,11-13].

Although the combination of the triple-negative phenotype and the pathologic features described above have come to be considered characteristic of *BRCA1*-associated breast cancers, approximately 10 to 36% of breast cancers that occur in *BRCA1 *mutation carriers (*BRCA1 *carriers) are, in fact, ER-positive (ER+) [4,6,8,14,15]. Relatively little is known about these *BRCA1*-associated ER+ breast cancers or about the factors that predict for the ER status of breast cancers that develop in these women.

As *BRCA1 *cancers are so often ER-, it has been suggested that ER negativity is intrinsic to *BRCA1 *cancers and reflects the cell of origin of these tumors [16]. Supporting this theory, Hosey and colleagues [17] have shown that transfection of the wild-type *BRCA1 *gene into HCC1937 cells, an ER- breast cancer cell line homozygous for the *BRCA1 *mutation, restores ER production. Likewise, knockdown of *BRCA1 *expression in the ER+ cell lines MCF-7 and T47D eliminates expression of ER. These investigators further showed that *BRCA1 *protein regulates the synthesis of ER through binding to the estrogen receptor-alpha gene promoter, *ESR1*. Liu and colleagues [18] proposed that *BRCA1 *may actually be required in the differentiation of ER- stem/progenitor cells to ER+ luminal cells. In prophylactic mastectomy specimens from women with germline *BRCA1 *mutations, breast tissue was found that appeared to be histologically normal, yet displayed loss of heterozygosity (LOH) for *BRCA1 *and was positive for the expression of the cancer stem cell marker, ALDH1 and negative for the expression of ER. This finding suggested that loss of *BRCA1 *may result in the accumulation of ER- breast stem cells, which are genetically unstable and more likely to undergo carcinogenesis.

If, in fact, ER negativity is intrinsic to *BRCA1 *cancers, this would raise the possibility that at least some *BRCA1 *ER+ cancers may be 'incidental', and not caused by a complete loss of *BRCA1 *function in the cancer cells.

It has been reported that ER+ breast cancers may be more common as *BRCA1 *carriers age [16]. If so, the frequency with which these ER+ breast cancers are encountered in clinical practice may increase as strategies for both prevention and treatment of the more common ER- breast cancers improve and mutation carriers live longer.

Given the paucity of information regarding ER+ breast cancers in *BRCA1 *mutation carriers, we undertook a study to: determine the clinical factors that predict for ER+ breast cancers in this population; compare the pathologic features of ER+ *BRCA1*-associated breast cancers with those of ER- *BRCA1*-associated breast cancers; and perform a case-control analysis to compare the pathologic features of ER+ *BRCA1*-associated breast cancers with those of ER+ sporadic breast cancers.

## Materials and methods

### Patient selection

Women with germline *BRCA1 *mutations who developed a first invasive breast cancer between 1979 and 2008 were retrospectively identified through the Cancer Risk and Prevention Programs at Beth Israel Deaconess Medical Center (BIDMC), Brigham and Women's Hospital/Dana-Farber Cancer Institute, and North Shore Medical Center. We identified 172 women with *BRCA1*-associated first invasive breast cancers (114 ER- and 58 ER+).

Among these 172 women, we were able to obtain pathologic material (H & E-stained sections and/or paraffin blocks) for 117 first invasive breast cancers (68 ER- and 49 ER+). Pathologic material was not available for cases diagnosed before 1986. For the case-control analysis, sporadic ER+ cancers (controls) were identified through the BIDMC tumor registry and consisted of women with a first invasive ER+ breast cancer and no family history of breast or ovarian cancer, matched on age and year of diagnosis (within three years) to the *BRCA1 *carriers with ER+ breast cancers (cases). Two *BRCA1*-associated cancers with 'low-positive' ER status were excluded from the case-control analysis because appropriate controls could not be identified. Three controls were identified for each mutation carrier except for three cases for which only two controls could be identified, resulting in a data set of 47 cases and 138 matched controls. Genetic testing records at BIDMC were reviewed to exclude potential control patients who had a positive test for a *BRCA1 *or *BRCA2 *mutation.

### Data collection

Clinical characteristics of *BRCA1 *carriers were abstracted from medical records, and included age at diagnosis, menopausal status at diagnosis, hormone use prior to diagnosis, Ashkenazi Jewish heritage, age at first live birth, smoking history, and alcohol use prior to diagnosis.

### Pathology review

Histologic sections of *BRCA1*-associated ER- and *BRCA1*-associated ER+ breast cancers were reviewed by the study pathologists blinded to the ER status of the tumor. Each cancer was scored for the following pathologic features: histologic type; Nottingham combined histologic grade, with each of the three components of grade (i.e., tubule formation, nuclear grade, and mitotic rate) recorded separately; presence of geographic necrosis or fibrotic focus; extent of lymphocytic infiltrate; and tumor margin characteristics (invasive or pushing). Histologic sections of the sporadic ER+ cancers were reviewed by the study pathologists and also assessed for each pathologic feature described above.

Information regarding ER status, assessed as part of the routine clinical evaluation, was abstracted from pathology reports. A biochemical method was used to determine the ER status between 1979 and 1992, and immunostaining was employed between 1993 and 2009. When information regarding the ER status for *BRCA1 *tumors was missing from the pathology report or when ER was reported as 'weak' or 'faint', paraffin blocks were re-cut and sections were immunostained for ER (rabbit monoclonal antibody SP1, Neomarkers, Fremont, CA, USA). Information regarding PR and HER2 status was also recorded for *BRCA1 *and control cancers.

### Statistical analysis

Analysis of the relation between ER status and clinical or pathologic factors used logistic regression, and analysis of the case-control study of ER+ *BRCA1 *and sporadic cancers used the conditional (matched) logistic regression. Both single covariate and step-up logistic regressions for multiple comparisons were performed, with two-sided *P *values from the likelihood ratio test. All continuous covariates were categorized as in the tables and used as binary variables in the models, with an additional binary variable for 'unknown' if the value of a covariate was unknown for at least two patients in each group (e.g. in ER+ and ER- patients). In reporting the final step-up model for each dataset, the *P *value for a covariate comes from the step at which it was added and the estimated risk ratio (RR) comes from the final step.

This study was approved by the institutional review boards of Dana Farber/Harvard Cancer Center and North Shore Medical Center.

## Results

### Relation between clinical factors and ER status of first breast cancers in *BRCA1 *mutation carriers

Table 1 summarizes the clinical characteristics of the 172 *BRCA1 *carriers and the results of single covariate logistic regression comparing these features between women with ER- and ER+ first breast cancers. Of these 172 patients, 34% (58) developed an ER+ first breast cancer. Two of these 58 cancers were 'weak' ER+ (with 1 to 10% of tumor cells showing ER expression). Of the 172 patients, 16.3% had ER measured using biochemical methods and 83.7% had ER measured using immunohistochemistry.

**Table 1:** Clinical characteristics of women with ER-negative and ER-positive *BRCA1*-associated breast cancers

Covariates	ER-		ER+		Logistic likelihood ratio test *P*
	
	n	%	N	%	
**All**	114	66	58	34	

**Menopausal status**					

Pre *	98	71	41	29	0.02

**Post**	15	47	17	53	

**Unknown**	1	100	0	0	

**Age of breast cancer**					

**Median (range)**	40 (27-73)		46 (29-72)		

**<40**	54	77	16	23	0.01

**40-49**	48	65	26	35	0.73

**≥ 50**	12	43	16	57	0.005

**Prior HRT**					

Yes *	11	58	8	42	0.42

**No**	101	67	50	33	

**Unknown**	2	100	0	0	

**Ashkenazi Jewish**					

Yes *	55	71	23	29	0.32

**No**	58	64	33	36	

Unknown *	1	33	2	67	

**First live birth before breast cancer?**					

Yes *	86	65	46	35	0.57

**No**	28	72	11	28	

**Unknown**	0	0	1	100	

**Age of first live birth (if before breast cancer)**					

**Median (range)**	27 (15-45)		27 (19-42)		

≥ 27 *	47	67	23	33	0.44

**<27**	39	63	23	37	

No or unknown live births before breast cancer *	28	70	12	30	

**Tobacco (pack years)**					

0 *	64	66	33	34	0.33

**>0**	31	74	11	26	

Unknown *	19	58	14	42	

**Alcohol**					

0-1 drinks/week *	65	68	30	32	0.75

**>1 drinks/week**	45	64	25	36	

Unknown *	4	57	3	43	

ER, estrogen receptor; HRT, hormone replacement therapy; -, negative; +, positive.

Percentages calculated by row

(*) indicates this level of the covariate was included in the single covariate model.

Age at breast cancer diagnosis was a significant predictor of ER status. The median age at breast cancer diagnosis was 40 years for women with an ER- cancer and 46 years for those with an ER+ cancer. Patients aged 50 years or older at diagnosis were significantly more likely to have an ER+ cancer compared with those younger than 50 years of age (16 of 28 = 57% vs 42 of 144 = 29%, *P *= 0.005). Conversely, patients younger than 40 years of age were significantly less likely to have an ER+ breast cancer compared with those aged 40 years or older (16 of 70 = 23% vs. 42 of 104 = 41%, *P *= 0.01).

In addition, pre-menopausal patients were significantly more likely to develop an ER- cancer compared with post-menopausal patients or those with unknown menopausal status (*P *= 0.02). Although only 29% of the breast cancers that developed in pre-menopausal *BRCA1 *carriers were ER+, 53% of the cancers in post-menopausal women were ER+.

In multiple covariate analysis, no covariate added significantly to the model after age 50 years or older was included. Although none of the women younger than 40 years of age at diagnosis were post-menopausal and only 14% of the women aged 40 to 49 years were post-menopausal, 21% of the women aged 50 years or older were pre-menopausal. None of the other clinical factors, including prior hormone use, Ashkenazi Jewish heritage, age at first live birth, smoking or alcohol use, predicted for ER status of the first breast cancer of these women.

### Comparison of pathologic features of ER- *BRCA1*-associated and ER+ *BRCA1*-associated breast cancers

Pathologic material was available for 49 of the 58 ER+ *BRCA1 *cancers and for 68 of the 114 ER- *BRCA1 *breast cancers. The distribution of cancers by age group was similar in the clinical and pathology data sets (Tables 1 and 2).

**Table 2:** Comparison of pathologic characteristics of ER-negative and ER-positive *BRCA1*-associated breast cancers

Covariates	ER-		ER+		Logistic likelihood ratio test *P*
		
	n	%	n	%	
**All**	68		49		

**Histologic type**					

Invasive ductal only *	67	99	37	76	<0.001

**Other**	1	1	12	24	

**Mixed ductal/lobular**	1		10		

**Tubular**	0		1		

**Lobular**	0		1		

**Histologic grade**					

3 *	66	97	20	41	<0.001

**1-2**	2	3	28	57	

**Unknown (combined with 1-2)**	0	0	1	2	

**Mitotic activity**					

>10 mitoses per 10 HPF *	64	94	11	22	<0.001

**0-10 mitoses per 10 HPF**	4	6	37	76	

**Unknown**	0	0	1	2	

**Margins**					

Invasive *	22	32	44	90	<0.001

**Pushing**	41	60	4	8	

**Unknown**	5	7	1	2	

**Lymphocytic infiltrate**					

Moderate/marked *	20	29	3	6	0.003

**None/mild**	46	68	45	92	

Unknown *	2	3	1	2	

**Geographic necrosis/fibrotic focus**					

Yes *	50	74	8	16	<0.001

**No**	15	22	39	80	

Unknown *	3	4	2	4	

**Age of breast cancer**					

**Median (range)**	39.5 (28-73)		45 (29-72)		

**<40**	34	50	15	31	0.03

**40-49**	27	40	20	41	0.91

**≥ 50**	7	10	14	29	0.01

ER, estrogen receptor; HPF, high-powered field; -, negative; +, positive.

Percentages calculated by column

(*) indicates this level of the covariate was included in the single covariate model

Table 2 compares the pathologic characteristics of the ER+ and ER-*BRCA1 *cancers. In single covariate models, ER+ *BRCA1 *cancers were found less often than ER- *BRCA1 *cancers to be of pure invasive ductal type (*P *< 0.001), to be histologic grade 3 (*P *< 0.001), to possess a high mitotic rate (>10 mitoses per 10 high powered field (HPF); *P *< 0.001), to have a moderate/marked lymphocytic infiltrate (*P *= 0.003), to have either geographic necrosis or a fibrotic focus (*P *< 0.001) or to have pushing (or unknown) margins (*P *< 0.001). Most of these differences remained significant even when limiting the comparison to histologic grade 3 *BRCA1 *ER+ and ER- cancers. In particular, grade 3 ER+ *BRCA1 *cancers less often had a high mitotic rate (*P *< 0.001), geographic necrosis/fibrotic focus (*P *= 0.002), or pushing/unknown margins (*P *< 0.001).

In a step-up logistic model, pathologic variables significantly predictive of a lower likelihood of having an ER+ breast cancer were high mitotic activity (RR 0.06, *P *< 0.001), geographic necrosis or fibrotic focus (RR 0.22, *P *= 0.02), and pushing/unknown margins (RR 0.24, *P *= 0.03). Of note, only 4% of ER+ *BRCA1 *breast cancers possessed all three of these features and 67% lacked all three features (compared with 50% and 3%, respectively, of ER- *BRCA1 *breast cancers). PR and HER2 status were collected for the *BRCA1 *breast cancers and are shown in Table 3.

**Table 3:** PR and HER2 status of *BRCA1*-associated breast cancers

	ER- *BRCA1 *cancers	ER+ *BRCA1 *cancers
	**n = 68**	**n = 49**

**PR positive**	0	40

HER2 +*	0	3 (1 IHC, 2 FISH)

HER2 negative**	0	34

HER2 equivocal^	0	2

HER2 unknown	0	1

**PR weak positive^^**	2	6

HER2 +*	0	1 (IHC)

HER2 -**	2	4

HER2 unknown	0	1

**PR negative**	59	1

HER2 +*	3 (IHC)	0

HER2 -**	48	1

HER2 equivocal^	1 (FISH)	0

HER2 unknown	7	

**PR unknown**	7	2

HER2 -**	6	2

HER2 unknown	1	0

ER, estrogen receptor; FISH, fluorescence *in situ *hybridization; HER, human epidermal growth factor receptor; IHC, immunohistochemistry; PR, progesterone receptor; -, negative; +, positive.

* HER2 positive: HER2:CEP17 ratio by FISH >2.2 or IHC 3+ (no FISH performed)

** HER2 negative: HER2:CEP17 ratio by FISH <1.8 or IHC <3+ (no FISH performed)

^HER2 equivocal: HER2:CEP17 ratio by FISH 1.8 to 2.2

^^ Weak PR positive: 1 to 10% cells show nuclear staining for PR

### Case-control analysis comparing pathologic features of *BRCA1*-associated ER+ breast cancers with ER+ sporadic breast cancers

The pathologic features of the 47 ER+ *BRCA1 *cancers and 138 ER+ sporadic cancers are shown in Table 4. Compared with ER+ sporadic cancers, ER+ *BRCA1 *cancers were more often of pure invasive ductal type (*P *= 0.03), more often had a high mitotic rate (>10 mitoses per 10 HPF, *P *= 0.03) and demonstrated a more limited spectrum of histologic types. In the step-up conditional logistic regression models, three variables were significantly more associated with ER+ *BRCA-1 *associated cancers than with ER+ sporadic controls: pure invasive ductal histology (RR 2.4, *P *= 0.03), 10 or more mitoses per 10 HPF (RR 5.0, *P *= 0.006), and absent or mild lymphocytic infiltrate (RR 10.2, *P *= 0.04).

**Table 4:** Comparison of pathologic features of ER-positive *BRCA1*-associated breast cancers and ER-positive sporadic breast cancers

**Covariates**	***BRCA1 *+**		**Sporadic**		
		
	**n**	**%**	**N**	**%**	**Logistic likelihood ratio test *P***
	
**All**	47	100	138	100	
	
**Mean age in years (range)**	46 (29-72)		46 (29-72)		
	
**Histologic type**					
	
**Invasive ductal only**	35	74	78	57	0.03
	
**All others**	12	26	60	43	
	
**Mixed ductal/lobular**	10	21	25	18	
	
**Lobular**	1	2	12	9	
	
**Mixed ductal + special type**	0	0	8*	6	
	
**Special type**	1^+^	2	15^++^	11	
	
**Histologic grade**					
	
**3**	18	38	34	25	0.10
	
**2**	15	32	69	50	0.39
	
**1**	13	28	35	25	0.69
	
**Unknown**	1	2	0	0	
	
**Mitoses/10 HPF**					
	
**>10**	9	19	9	7	0.03
	
**6-10**	12	26	31	22	0.67
	
**0-5**	25	53	98	71	0.03
	
**Unknown**	1	2	0	0	
	
**Margins**					
	
**Invasive**	43	91	134	97	0.12
	
**Pushing**	3	6	2	1	0.09
	
**Unknown**	1	2	2	1	
	
**Lymphocytic infiltrate**					
	
**Moderate/marked**	2	4	17	12	0.08
	
**None/mild**	44	94	121	88	
	
**Unknown**	1	2	0	0	
	
**Geographic necrosis/fibrotic focus**					
	
**Yes**	7	15	9	7	0.09
	
**No**	38	81	129	93	
	
**Unknown**	2	4	0	0	

ER, estrogen receptor; HPF, high powered field.

Percentages calculated by column

* Mixed ductal plus: mucinous (n = 4), invasive micropapillary (n = 2), invasive papillary (n = 1), tubular (n = 1)

+ tubular (n = 1)

++ tubular (n = 6), mucinous (n = 5), invasive micropapillary (n = 2), invasive cribriform (n = 1), mixed invasive

micropapillary/mucinous (n = 1)

Comparison of PR and HER2 status between ER+ *BRCA1 *breast cancers and sporadic controls was not possible due to the unavailability of data for many of the controls.

Twenty-nine of the 138 patients with ER+ sporadic breast cancers (21%) had undergone genetic testing at BIDMC and none was found to have a *BRCA1 *or *BRCA*2 mutation. Some of the other women with ER+ sporadic breast cancers may have undergone genetic testing at other institutions, but that information was not available.

## Discussion

The results of this study suggest that *BRCA1 *carriers who are older at the time of diagnosis of their first invasive breast cancer are more likely to have an ER+ breast cancer than are *BRCA1 *carriers who are younger at diagnosis. Menopausal status was also a predictor of ER positivity, with ER+ breast cancers being more common in post-menopausal carriers. However, this difference was not significant in multiple covariate analysis, perhaps because of the confounding between menopausal status and age. In particular, only 14% of *BRCA1 *carriers younger than age 50 years in our study were post-menopausal. As mutation carriers increasingly become surgically menopausal at younger ages it will be important to determine the relative contributions of age and menopausal status for predicting ER status of the breast cancers that develop in this population. Our data are consistent with those of Foulkes and colleagues [16] who also found an increase in ER+ breast cancers with increasing age among *BRCA1 *mutation carriers. These investigators noted that this increase in ER positivity paralleled that seen in breast cancers that develop in non-mutation carriers. They did not study the effect of menopausal status on ER status of these cancers. The observation that *BRCA1 *mutation carriers who are older or post-menopausal at the time of diagnosis of breast cancer are more likely to have an ER+ breast cancer may help to define a population of *BRCA1 *mutation carriers for whom estrogen-modifying agents will be particularly effective.

Of the *BRCA1 *cancers in this series, 34% were ER+. This is consistent with the 31% frequency of ER+ *BRCA1 *breast cancers recently reported in the retrospective series by Atchley and colleagues [8].

Our comparison of the pathologic features of ER+ and ER- *BRCA1 *cancers revealed that the ER+ cancers less often had features typically associated with *BRCA1 *cancers, such as high mitotic rate, pushing margins, marked lymphocytic infiltrate, and geographic necrosis/fibrotic focus. These differences were not due to differences in histologic grade, because most remained significant when only high-grade ER+ and ER- cancers were compared. Although previous studies have noted that ER- *BRCA1 *cancers are more likely to be high-grade invasive ductal carcinomas compared with ER+ *BRCA1 *cancers, this is the first report to our knowledge analyzing the relation of ER status to other pathologic features that have come to be considered to be *BRCA*-related.

The differences in pathologic features between ER+ and ER- *BRCA1 *cancers raise the possibility that at least some *BRCA1 *ER+ cancers may be 'incidental', and not caused by a complete loss of *BRCA1 *function in the cancer cells. In order to address the issue of whether ER+ *BRCA1 *cancers are more akin to sporadic ER+ breast cancers than to ER- *BRCA1 *cancers, we performed a case-control analysis in which the pathologic features of these tumors were compared with those of a control group of ER+ sporadic breast cancers. We found that *BRCA1*-associated ER+ cancers had a much more limited distribution of histologic types and were significantly more often pure invasive ductal carcinomas with a high mitotic rate than ER+ sporadic cancers.

There are several possible explanations for our observation that the histopathology of ER+ *BRCA1 *breast cancers differs significantly from both ER- *BRCA1 *cancers as well as ER+ sporadic breast cancers. First, it may be that although some ER+ *BRCA1 *breast cancers develop from complete loss of *BRCA1 *function, others still have intact *BRCA1 *function resulting in tumors that as a group have phenotypic features that are intermediate between ER- *BRCA1 *and ER+ sporadic breast cancers. The issue of whether ER+ *BRCA1*-associated breast cancers demonstrate LOH for the wild-type (wt) *BRCA1 *allele has been investigated. In this regard, Manié and colleagues recently found 4 of 19 ER+ *BRCA1*-associated breast cancers did not show loss of the wt *BRCA1 *allele [19]. King and colleagues [20] demonstrated that 11 of 22 *BRCA1*-associated invasive breast cancers did not show LOH for wt *BRCA1*; no mention of ER status was included in their study. The results of these studies are difficult to compare because of differences in patient populations and molecular methodology. Nonetheless, taken together the results of these two studies raise the possibility that not all *BRCA1*-associated breast cancers exhibit complete loss of *BRCA1 *function. However, the frequency of this phenomenon, particularly for ER+ *BRCA1 *cancers, remains to be more clearly defined.

It is also possible that no breast cancer that develops in a *BRCA1 *mutation carrier is really 'incidental' or sporadic, even if LOH of wt *BRCA1 *does not exist. Haploinsufficiency of *BRCA1*, which exists in the *BRCA1 *heterozygous state, has been shown to have demonstrable effects on the breast tissue of *BRCA1 *carriers. Normal breast tissue from *BRCA1 *carriers has been shown to grow abnormally in three-dimensional mammosphere cultures (even though 75% of cells show retention of *BRCA1 *heterozygosity) [21], and express increased aromatase [22] compared with reduction mammoplasty specimens from non-mutation carriers. Likewise, MCF-7 cells with *BRCA1 *haploinsuffiency demonstrate decreased efficiency in homologous recombination [23]. Haploinsufficiency of *BRCA1 *may predispose both to the development of breast cancer as well as to a more limited histopathologic profile. Finally, if loss of *BRCA1 *function does exist in the majority of ER+ *BRCA1 *breast cancers, it is possible that ER+ and ER- *BRCA1 *cancers originate from different cells of origin (e.g. early progenitor cell vs stem cell) leading to different phenotypic expressions. The cell of origin for *BRCA1*-associated breast cancers is still being determined [24,25].

One of the strengths of this study is that the pathologic features of all cancers in this study, both *BRCA1 *and control, were reviewed by two dedicated breast pathologists (SJS and LCC). It should be noted that more of the *BRCA1 *ER- breast cancers identified were unavailable for pathologic review. Although 34% of the cases in the clinical analysis were ER+, 42% of the cancers reviewed pathologically were ER+. Given the uniformity of many of the pathologic features of the *BRCA1 *ER- breast cancers in this study, we think it is unlikely that this substantially affected the major findings of our study.

One potential limitation of the case-control study is that *BRCA1 *and *2 *genetic testing information was not available for all of the women with sporadic cancers. However, as *BRCA1 *and *BRCA2 *cancers comprise only 5 to 10% of all cancers and potential controls were excluded if a family history of breast or ovarian cancer was noted in the medical record, it seems very unlikely that more than a few of the 'control' cases had germline *BRCA1 *mutations. We intentionally chose controls from the general hospital population rather than from those who tested negative for *BRCA *mutations through the genetic testing clinic. Thus, our group of controls is more likely to represent sporadic breast cancers than those identified through a genetic testing program, many of whom may have inherited breast cancers, although not through a germline *BRCA1 *or *2 *mutation.

## Conclusions

In conclusion, the results of this study indicate that *BRCA1 *carriers who are older at the time of invasive breast cancer diagnosis are more likely to have ER+ breast cancers than younger *BRCA1 *carriers. Furthermore, ER+ *BRCA1 *breast cancers appear to be pathologically 'intermediate' between ER- *BRCA1 *cancers and ER+ sporadic cancers, thus comprising a unique group. These observations raise the possibility that either some of the ER+ *BRCA1 *cancers are incidental (i.e. not *BRCA1*-related), or that there is a unique mechanism by which they develop. Given the availability of new and effective therapies that exploit the defect in homologous recombination, which exists in *BRCA1*-related cancers such as poly (ADP-ribose) polymerase (PARP) inhibitors [26] and Cisplatin [27], it will become increasingly important to determine whether the pathways leading to ER+ *BRCA1 *breast cancers are similar to those that result in ER- *BRCA1 *cancers and whether these new therapies are likely to be effective in ER+ *BRCA1 *cancers. Toward this end, a detailed immunophenotypic and molecular analysis of the ER+ *BRCA1 *cancers is currently underway.

## Abbreviations

BIDMC: Beth Israel Deaconess Medical Center; ER: estrogen receptor; H & E: hematoxylin and eosin; HER: human epidermal growth factor receptor; HPF: high powered field; LOH: loss of heterozygosity; PR: progesterone receptor; RR: risk ratio; wt: wild-type; -: negative; +: positive.

## Competing interests

The authors declare that they have no competing interests.

## Authors' contributions

NT wrote the manuscript with SJS, shared in study design, oversaw the collection of data and pathology material, and reviewed data. YW and JK aided in collection and review of pathology material. LC with SS reviewed all study pathology material. HL and RG performed statistical analysis. AC collected clinical data on study patients. BG, KF, and KS provided data management. KK, RL, PDR and DS identified cases and supplied pathologic material. JEG helped in study design and identification of study material. SJS shared in study design, reviewed with LC all pathology material, reviewed data and helped in writing the manuscript.

## References

[B1] Eisinger F Stoppa-Lyonnet D Longy M Kerangueven F Noguchi T Bailly C Vincent-Salomon A Jacquemier J Birnbaum D Sobol H Germ line mutation at BRCA1 affects the histoprognostic grade in hereditary breast cancer Cancer Res 1996 56 471 474 8564955

[B2] Karp SE Tonin PN Begin LR Martinez JJ Zhang JC Pollak MN Foulkes WD Influence of BRCA1 mutations on nuclear grade and estrogen receptor status of breast carcinoma in Ashkenazi Jewish women Cancer 1997 80 435 441 10.1002/(SICI)1097-0142(19970801)80:3<435::AID-CNCR11>3.0.CO;2-Y 9241077

[B3] Robson M Gilewski T Haas B Levin D Borgen P Rajan P Hirschaut Y Pressman P Rosen PP Lesser ML Norton L Offit K BRCA-associated breast cancer in young women J Clin Oncol 1998 16 1642 1649 9586873 10.1200/JCO.1998.16.5.1642

[B4] Verhoog LC Brekelmans CT Seynaeve C Bosch LM van den Dahmen G van Geel AN Tilanus-Linthorst MM Bartels CC Wagner A Ouweland A van den Devilee P Meijers-Heijboer EJ Klijn JG Survival and tumour characteristics of breast-cancer patients with germline mutations of BRCA1 Lancet 1998 351 316 321 10.1016/S0140-6736(97)07065-7 9652611

[B5] Quenneville LA Phillips KA Ozcelik H Parkes RK Knight JA Goodwin PJ Andrulis IL O'Malley FP HER-2/neu status and tumor morphology of invasive breast carcinomas in Ashkenazi women with known BRCA1 mutation status in the Ontario Familial Breast Cancer Registry Cancer 2002 95 2068 2075 10.1002/cncr.10949 12412159

[B6] Lakhani SR Vijver MJ Van De Jacquemier J Anderson TJ Osin PP McGuffog L Easton DF The pathology of familial breast cancer: predictive value of immunohistochemical markers estrogen receptor, progesterone receptor, HER-2, and p53 in patients with mutations in BRCA1 and BRCA2 J Clin Oncol 2002 20 2310 2318 10.1200/JCO.2002.09.023 11981002

[B7] Foulkes WD Stefansson IM Chappuis PO Begin LR Goffin JR Wong N Trudel M Akslen LA Germline BRCA1 mutations and a basal epithelial phenotype in breast cancer J Natl Cancer Inst 2003 95 1482 1485 14519755 10.1093/jnci/djg050

[B8] Atchley DP Albarracin CT Lopez A Valero V Amos CI Gonzalez-Angulo AM Hortobagyi GN Arun BK Clinical and pathologic characteristics of patients with BRCA-positive and BRCA-negative breast cancer J Clin Oncol 2008 26 4282 4288 10.1200/JCO.2008.16.6231 18779615 PMC6366335

[B9] Armes JE Egan AJ Southey MC Dite GS McCredie MR Giles GG Hopper JL Venter DJ The histologic phenotypes of breast carcinoma occurring before age 40 years in women with and without BRCA1 or BRCA2 germline mutations: a population-based study Cancer 1998 83 2335 2345 10.1002/(SICI)1097-0142(19981201)83:11<2335::AID-CNCR13>3.0.CO;2-N 9840533

[B10] Lakhani SR Jacquemier J Sloane JP Gusterson BA Anderson TJ Vijver MJ van de Farid LM Venter D Antoniou A Storfer-Isser A Smyth E Steel CM Haites N Scott RJ Goldgar D Neuhausen S Daly PA Ormiston W McManus R Scherneck S Ponder BA Ford D Peto J Stoppa-Lyonnet D Bignon YJ Struewing JP Spurr NK Bishop DT Klijn JG Devilee P Multifactorial analysis of differences between sporadic breast cancers and cancers involving BRCA1 and BRCA2 mutations J Natl Cancer Inst 1998 90 1138 1145 10.1093/jnci/90.15.1138 9701363

[B11] Laakso M Loman N Borg A Isola J Cytokeratin 5/14-positive breast cancer: true basal phenotype confined to BRCA1 tumors Mod Pathol 2005 18 1321 1328 10.1038/modpathol.3800456 15990899

[B12] Lakhani SR Reis-Filho JS Fulford L Penault-Llorca F Vijver M van der Parry S Bishop T Benitez J Rivas C Bignon YJ Chang-Claude J Hamann U Cornelisse CJ Devilee P Beckmann MW Nestle-Kramling C Daly PA Haites N Varley J Lalloo F Evans G Maugard C Meijers-Heijboer H Klijn JG Olah E Gusterson BA Pilotti S Radice P Scherneck S Sobol H Prediction of BRCA1 status in patients with breast cancer using estrogen receptor and basal phenotype Clin Cancer Res 2005 11 5175 5180 10.1158/1078-0432.CCR-04-2424 16033833

[B13] Arnes JB Brunet JS Stefansson I Begin LR Wong N Chappuis PO Akslen LA Foulkes WD Placental cadherin and the basal epithelial phenotype of BRCA1-related breast cancer Clin Cancer Res 2005 11 4003 4011 10.1158/1078-0432.CCR-04-2064 15930334

[B14] Johannsson OT Idvall I Anderson C Borg A Barkardottir RB Egilsson V Olsson H Tumour biological features of BRCA1-induced breast and ovarian cancer Eur J Cancer 1997 33 362 371 10.1016/S0959-8049(97)89007-7 9155518

[B15] Loman N Johannsson O Bendahl PO Borg A Ferno M Olsson H Steroid receptors in hereditary breast carcinomas associated with BRCA1 or BRCA2 mutations or unknown susceptibility genes Cancer 1998 83 310 319 10.1002/(SICI)1097-0142(19980715)83:2<310::AID-CNCR15>3.0.CO;2-W 9669814

[B16] Foulkes WD Metcalfe K Sun P Hanna WM Lynch HT Ghadirian P Tung N Olopade OI Weber BL McLennan J Olivotto IA Begin LR Narod SA Estrogen receptor status in BRCA1- and BRCA2-related breast cancer: the influence of age, grade, and histological type Clin Cancer Res 2004 10 2029 2034 10.1158/1078-0432.CCR-03-1061 15041722

[B17] Hosey AM Gorski JJ Murray MM Quinn JE Chung WY Stewart GE James CR Farragher SM Mulligan JM Scott AN Dervan PA Johnston PG Couch FJ Daly PA Kay E McCann A Mullan PB Harkin DP Molecular basis for estrogen receptor alpha deficiency in BRCA1-linked breast cancer J Natl Cancer Inst 2007 99 1683 1694 10.1093/jnci/djm207 18000219 PMC6485437

[B18] Liu S Ginestier C Charafe-Jauffret E Foco H Kleer CG Merajver SD Dontu G Wicha MS BRCA1 regulates human mammary stem/progenitor cell fate Proc Natl Acad Sci USA 2008 105 1680 1685 10.1073/pnas.0711613105 18230721 PMC2234204

[B19] Manie E Vincent-Salomon A Lehmann-Che J Pierron G Turpin E Warcoin M Gruel N Lebigot I Sastre-Garau X Lidereau R Remenieras A Feunteun J Delattre O de The H Stoppa-Lyonnet D Stern MH High frequency of TP53 mutation in BRCA1 and sporadic basal-like carcinomas but not in BRCA1 luminal breast tumors Cancer Res 2009 69 663 671 10.1158/0008-5472.CAN-08-1560 19147582

[B20] King TA Li W Brogi E Yee CJ Gemignani ML Olvera N Levine DA Norton L Robson ME Offit K Borgen PI Boyd J Heterogenic loss of the wild-type BRCA allele in human breast tumorigenesis Ann Surg Oncol 2007 14 2510 2518 10.1245/s10434-007-9372-1 17597348

[B21] Burga LN Tung NM Troyan SL Bostina M Konstantinopoulos PA Fountzilas H Spentzos D Miron A Yassin YA Lee BT Wulf GM Altered proliferation and differentiation properties of primary mammary epithelial cells from BRCA1 mutation carriers Cancer Res 2009 69 1273 1278 10.1158/0008-5472.CAN-08-2954 19190334 PMC3041511

[B22] Chand AL Simpson ER Clyne CD Aromatase expression is increased in BRCA1 mutation carriers BMC Cancer 2009 9 148 10.1186/1471-2407-9-148 19445691 PMC2689243

[B23] Cousineau I Belmaaza A BRCA1 haploinsufficiency, but not heterozygosity for a BRCA1-truncating mutation, deregulates homologous recombination Cell Cycle 2007 6 962 971 17404506 10.4161/cc.6.8.4105

[B24] Lim E Vaillant F Wu D Forrest NC Pal B Hart AH Asselin-Labat ML Gyorki DE Ward T Partanen A Feleppa F Huschtscha LI Thorne HJ Fox SB Yan M French JD Brown MA Smyth GK Visvader JE Lindeman GJ Aberrant luminal progenitors as the candidate target population for basal tumor development in BRCA1 mutation carriers Nat Med 2009 15 907 913 10.1038/nm.2000 19648928

[B25] Ginestier C Liu S Wicha MS Getting to the root of BRCA1-deficient breast cancer Cell Stem Cell 2009 5 229 230 10.1016/j.stem.2009.08.007 19733528

[B26] Tutt A RM Garber JE Domchek S Audeh MW Weitzel JN Friedlander M Carmichael J Phase II trial of the oral PARP inhibitor olaparib in BRCA-deficient advanced breast cancer J Clin Oncol 2009 27 18s Abstract CRA501 10.1200/JCO.2009.22.4626

[B27] Gronwald J BT Huzarski T Dent R Bielick V Zuziak D Wisniowski R Lubinski J Narod S Neoadjuvant therapy with cisplatin in BRCA1-positive breast cancer patients J Clin Oncol 2009 27 15s Abstract 502 10.1200/JCO.2008.21.7695

